# Natural biopolymer scaffold for meniscus tissue engineering

**DOI:** 10.3389/fbioe.2022.1003484

**Published:** 2022-09-30

**Authors:** Yachen Peng, Meng Lu, Zhongsheng Zhou, Chenyu Wang, Enbo Liu, Yanbo Zhang, Tong Liu, Jianlin Zuo

**Affiliations:** ^1^ Department of Orthopedics, China-Japan Union Hospital of Jilin University, Changchun, China; ^2^ Department of Nursing, The First Bethune Hospital of Jilin University, Changchun, China; ^3^ Scientific Research Center, China-Japan Union Hospital of Jilin University, Changchun, China

**Keywords:** sport medicine, tissue engineering, meniscus, natural biopolymer, clinic transition

## Abstract

Meniscal injuries caused by trauma, degeneration, osteoarthritis, or other diseases always result in severe joint pain and motor dysfunction. Due to the unique anatomy of the human meniscus, the damaged meniscus lacks the ability to repair itself. Moreover, current clinical treatments for meniscal injuries, including meniscal suturing or resection, have significant limitations and drawbacks. With developments in tissue engineering, biopolymer scaffolds have shown promise in meniscal injury repair. They act as templates for tissue repair and regeneration, interacting with surrounding cells and providing structural support for newly formed meniscal tissue. Biomaterials offer tremendous advantages in terms of biocompatibility, bioactivity, and modifiable mechanical and degradation kinetics. In this study, the preparation and composition of meniscal biopolymer scaffolds, as well as their properties, are summarized. The current status of research and future research prospects for meniscal biopolymer scaffolds are reviewed in terms of collagen, silk, hyaluronic acid, chitosan, and extracellular matrix (ECM) materials. Overall, such a comprehensive summary provides constructive suggestions for the development of meniscal biopolymer scaffolds in tissue engineering.

## 1 Introduction

Meniscal tear is one of the commonplace injuries of the knee joint, which is often caused by trauma, degeneration, and osteoarthritis (Beaufils et al., 2017; Culvenor et al., 2019; Kopf et al., 2020). Partial or total meniscectomy and meniscus suture repair are commonly used to treat meniscus injuries and knee pain, but in terms of long-term prognosis, therapeutic effects are often limited (van de Graaf et al., 2018; Abram et al., 2020), and there are certain complications of treatment, such as cartilage injuries (Venjakob et al., 2019). Therefore, finding a safe and effective alternative therapy to repair meniscal tears is critical for orthopedists.

The repair capacity of the meniscus is related to the degree of vascular infiltration and structure. Compared to lateral edge of the meniscus, the medial edge lacks vascularity and therefore meniscus is less capable of self-repair. The complex anisotropic structure of meniscus increases the difficulty of self-repair. To adapt to compression, stretch, and torsion stresses of the knee from all directions at rest or during sports, the collagen arrangement of meniscus shows a gradient change. The continuous development of tissue engineering has made it possible to repair the meniscus perfectly. Tissue engineering can mimic the original natural tissue and stimulate the body’s regenerative potential to promote the growth of tissue into the scaffold and complete the gradual replacement to form functional tissue.

**FIGURE 1:**
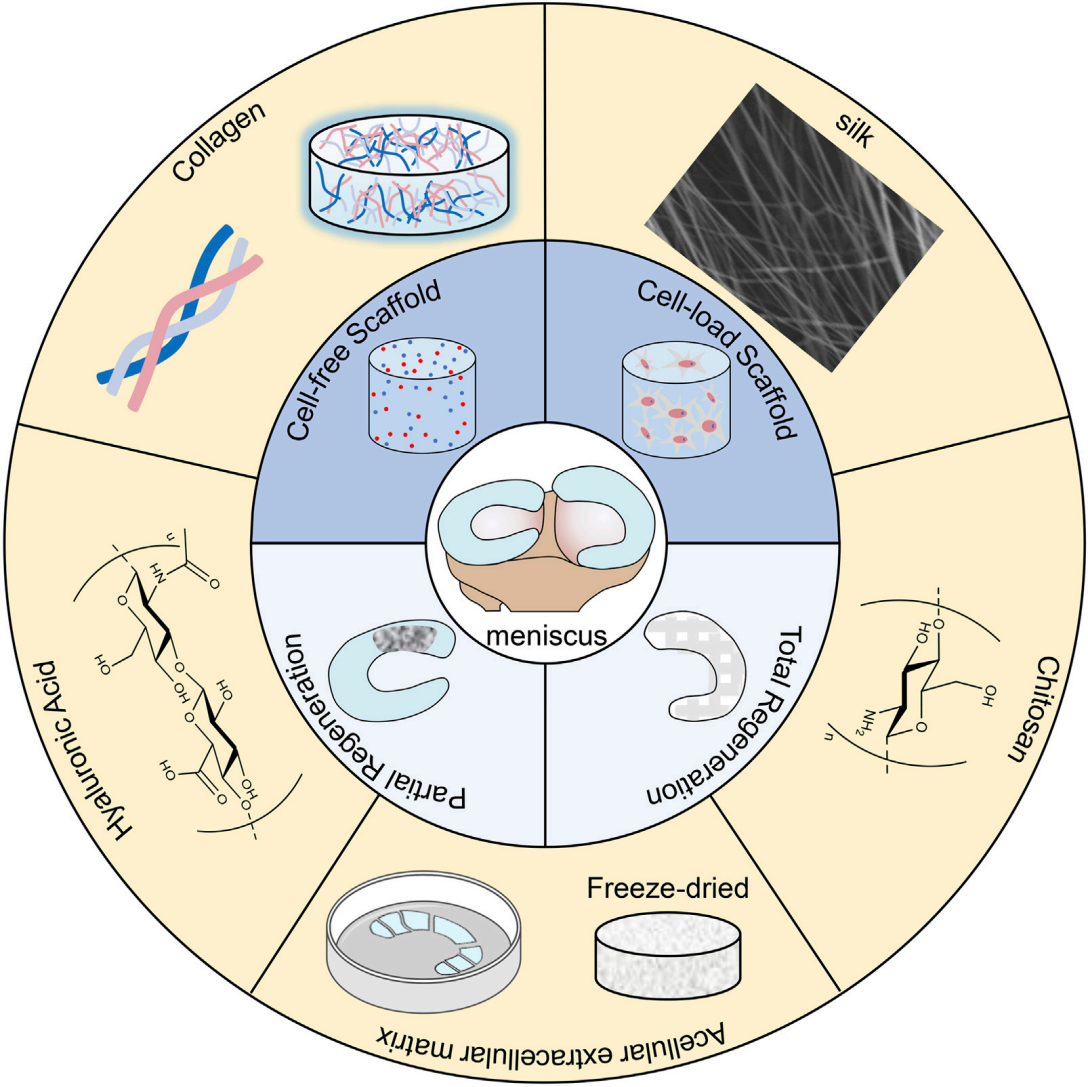
Schematic illustration of natural materials for meniscus tissue engineering.

Recent researches have found that biopolymer scaffolds have favorable development foreground in the repair of a variety of tissue injuries (Sandri et al., 2019; Stoppel et al., 2015). They act as patterns for tissue repair and regeneration, interacting with surrounding cells and providing structural support for newly formed tissue (Hsu et al., 2016). Polymers of natural origin, such as collagen, silk, hyaluronic acid, chitosan, and extracellular matrix, are used for tissue engineering due to their good biocompatibility and bioactivity as well as their adjustable mechanical and degradation properties (Figure 1) (Stoppel et al., 2015; Wubneh et al., 2018; Zhang et al., 2019). And biopolymer scaffolds are receiving soaring attention from researchers in the treatment of meniscal injuries (Lin et al., 2017). For example, a collagen meniscus implant (CMI) made from type I collagen from the bovine achilles tendon has been used in clinical treatment (Stone et al., 1997). CMI provides good and consistent clinical effects, especially in terms of knee function and pain, with a low incidence of complications and reoperation (Grassi et al., 2014). However, meniscal scaffolds still have many problems to be solved (Veronesi et al., 2021), such as the lack of gradient bionic and insufficient mechanical properties. Although natural material scaffolds have many advantages in repairing meniscus, they can still be optimized in terms of gradient bionic structure, anti-inflammatory, and repair synergy to improve the repair efficiency (Hao et al., 2021). There is a wide variety of biopolymer scaffolds and most of them are still in the experimental stage, and we still lack a systematic understanding of their investigation. In this review, we refer to the research literature of recent years to systematically elucidate biopolymer meniscal scaffolds from various aspects.

## 2 Basic properties of the meniscus

### 2.1 Vascular, neural, and basic anatomy of the meniscus

**FIGURE 2:**
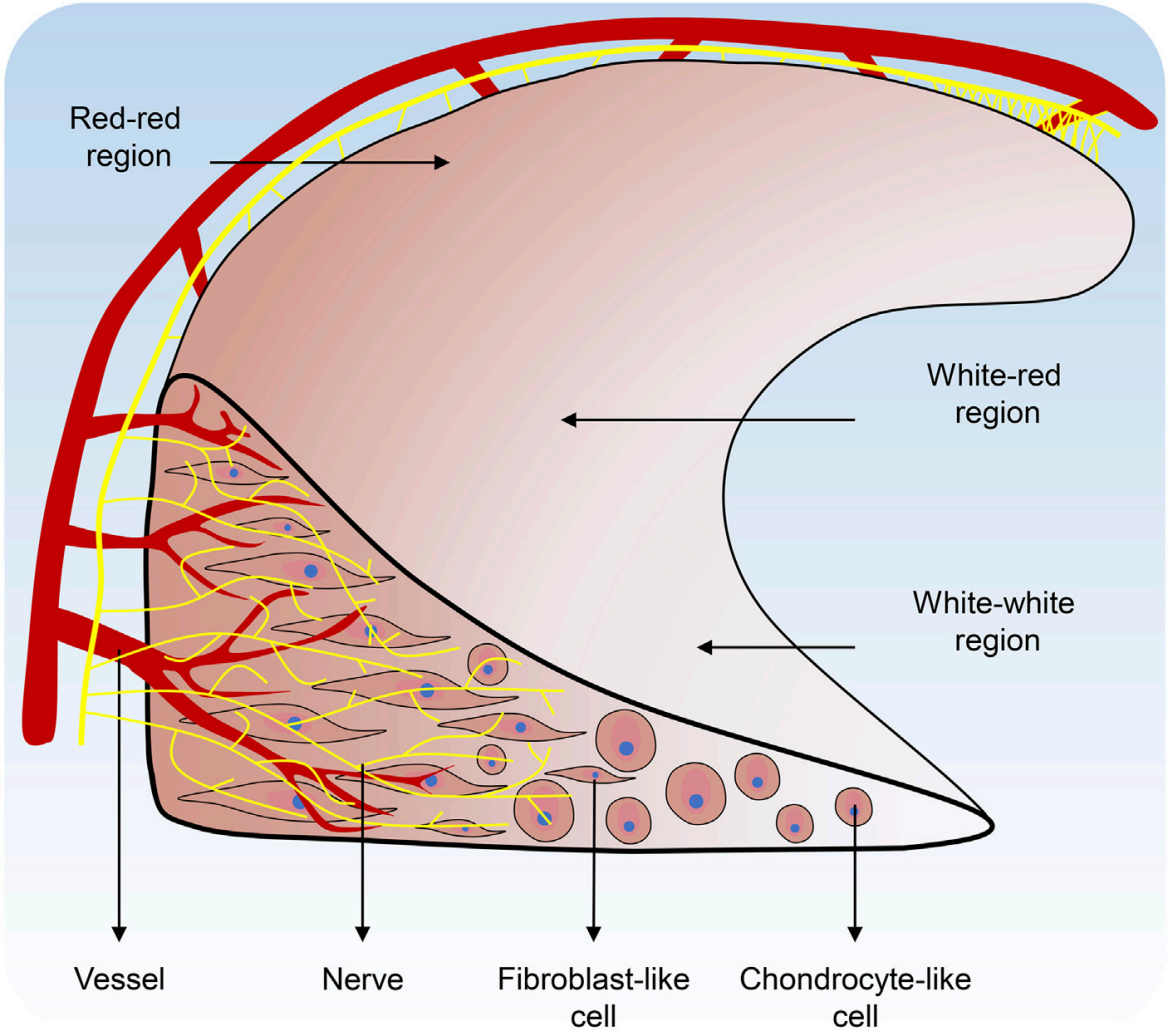
Regional variations in vascularization, nerve distribution, and cell populations of the Meniscus.

The meniscus is unique in that it manifests as discrete cartilage-like tissue during embryonic life and subsequently depends on its location to be shaped by different external environmental stimuli. Ultimately, the vascular formation and innervation would be affected to develop into different tissue stratum. Signal transduction, vascular formation, innervation, stem cell origin, and location in the body all produce subtle tissue development differences. These distinctions guide the subpopulations of cells that will ultimately make up the tissue (Figure 2).

The capillary plexus surrounding the meniscus, which originates from the joint capsule and synovial tissue, provides 10–25% of the blood supply to the meniscus and is confined to the peripheral third of the meniscus (Day et al., 1985). The meniscus anterior and posterior horn attachments are covered by vascular synovial tissue, which provides an adequate blood supply to the meniscus (Arnoczky & Warren, 1982). The meniscus can be divided into three regions: the outer red-red region (vascular/nerve region), the inner white-white region (non-vascular/non-nerve region), and the transitional red-white region.

The nerves and mechanoreceptors of the meniscus are mainly located in the anterior and posterior horns of the meniscus and the lateral two-thirds of the body. The medial third of the meniscus is thin and lacks nerves (Gray, 1999). This particular nerve arrangement in the meniscus excites the internal mechanical drive system for proper joint alignment and biomechanical function when the meniscus is subjected to forces, resulting in a degree of pressure and tension on the meniscal surface (Zimny et al., 1988).

There is a significant difference in size between the lateral and medial meniscus in humans. The medial meniscus is C-shaped and covers 51%–74% of the area of the medial tibia plateau. The medial meniscus is slightly thinner in volume (Fox et al., 2015). It is more widely open to the medial intercondylar notch and has a relatively flat tibiofemoral articular surface (Renström & Johnson, 1990). The lateral meniscus is rounded and smaller but more flexible than the medial meniscus. It is located in the lateral intercondylar fossa of the tibial plateau and covers 75%–93% of the lateral tibial plateau (Kummer, 1987; Masouros et al., 2008).

### 2.2 Matrix microenvironment and biochemistry of the meniscus

The meniscus comprises cells and an extracellular matrix of collagen, proteoglycan, matrix glycoproteins, and elastin. The outer third of the Meniscus is dominated by fibroblasts and the inner third is by chondrocytes. The middle third of the meniscus has fibro chondrocytes (Markes et al., 2020). Collagen is the main fibrous component of the meniscus, mainly resists the tensile stress experienced by the meniscus, and its content varies greatly in the different regions of the meniscus. In the red-red region, type I collagen accounts for 80% of the dry weight (Cheung, 1987). The collagen fibers in the main body of the meniscus are arranged in circular patterns, allowing pressure loads to be distributed, while the surface and middle of the meniscus are radially distributed to resist longitudinal tears caused by external forces (Beaupré et al., 1986). Radial fibers are also present in the deeper regions of the meniscus, where they hold the circumferential fibers in place and maintain structural integrity (Shen et al., 2022). In the white-white region, collagen is composed of only Col II (60%) and Col I (40%) (Fox et al., 2012; Bansal et al., 2020).

**FIGURE 3:**
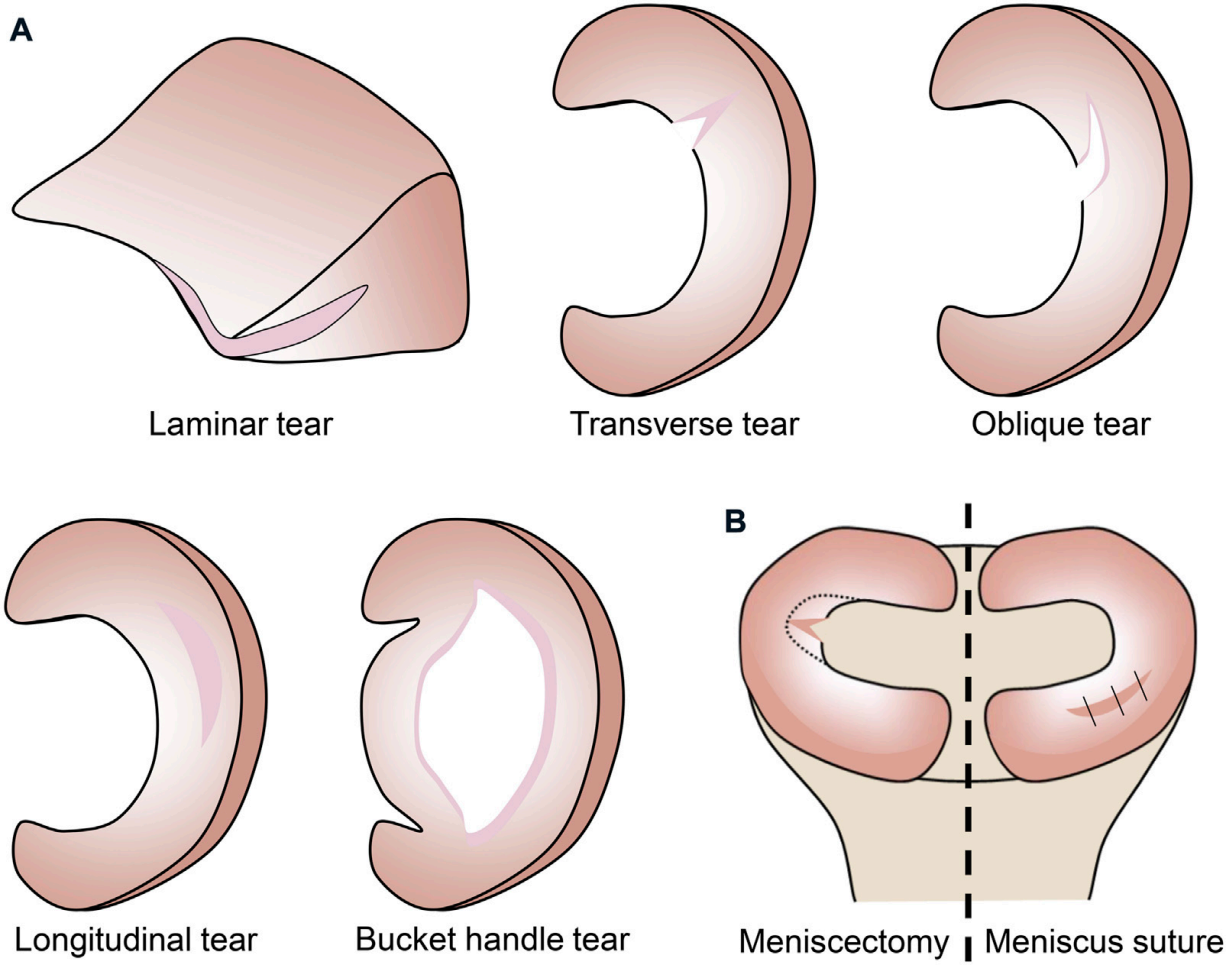
**(A)** Classification of meniscus injuries. **(B)** Treatment of meniscus injuries.

Therefore, the meniscus is an anisotropic structure, which results in a low capacity for self-repair and the site of injury can influence the choice of treatment options for the orthopedic surgeon. According to the mechanism of injury, meniscal injuries can be classified as laminar, transverse, longitudinal, bucket handle, and oblique tears (Figure 3A). Only injuries that are limited to the vascular red-red and red-white zones are considered to have repair potential with meniscal suturing. Injuries involving the avascular white-white zone have low repair capacity, and orthopedic surgeons often opt for meniscectomy (Figure 3B) to address the patient’s pain. However, when the cartilage loses the protection of the meniscus, degenerative changes may occur and eventually the inevitable development of osteoarthritis.

## 3 Natural materials used for meniscus tissue engineering

Tissue engineering is the implantation of a scaffold loaded with cytokines or cells into tissue damage, and the gradual transformation of the scaffold into biological tissues, to achieve the purpose of repairing the damage. Scaffolds are the carriers of cytokines and cells. Therefore, as the key to tissue engineering, the selection of scaffold materials is very important. Compared with synthetic materials, natural materials have many significant advantages (Table 1), such as good biocompatibility and biodegradability, a structure similar to tissues, and the most important biological inducing activity. For the repair of the meniscus, good biocompatibility and degradability can make the material or the degradation products of the material not produce an inflammatory response, and the scaffold can be degraded as the tissue grows in. The structure similar to the tissue allows the normal tissue around the injury to better integrate with the repair tissue. The most important bio-inducing activity can maintain the consistency of the regenerated tissue with the original tissue, to restore the function perfectly.

**TABLE 1:** Natural materials used for meniscus tissue engineering.

Materials	Processing technology	Repair area	Comparator	Loaded impact factors	Loaded cells	Results	References
Collagen	Electrospinning	*In vitro* culture	Scaffold with hydrogel or without hydrogel, with different cells	TGF-β1, TGF-β3	Meniscus cell, BMSC, synovial cell, cell from the IPFP	Collagen scaffolds with hydrogels loaded with IPFP cells yielded the highest cell densities with greater deposition of Col I and the highest mechanical properties compared to other cells.	Baek et al. (2018)
	Electrospinning	*Ex vivo* repair model	Collagen scaffold with meniscus cells	-	Meniscus cell	Cell-seeded collagen scaffolds resulted in better integration of new tissue with native tissue.	Baek et al. (2016)
	Freeze-drying	Partial meniscus repair	Intra-articular injections of vehicle or gefitinib	gefitinib	-	Intra-articular injection of gefitinib and implantation of a collagen scaffold enhanced meniscal regeneration.	Pan et al. (2017)
	Chemical crosslinking	*In vitro* culture	Scaffold with different PRP or whole blood	PRP	Meniscus cell	PRP has a higher effect on meniscus cell growth and gene expression than whole blood	Howard et al. (2014)
	Photocrosslinking	Partial meniscus repair	Cells expanded with conditioned medium or growth medium	TGF-β3	TMSC	Chondrogenic induced cells in the scaffold have more cell proliferation, GAG and collagen deposition for the best meniscal repair	(Heo et al., 2016; Koh et al., 2017)
Silk	Salt porogen leaching, freeze-drying	*In vitro* culture	Different layers of meniscal scaffold	-	Fibroblasts at the periphery and chondrocytes at the scaffold center	Chondrocytes in the inner region enhanced Col I and Col II production, and fibroblasts in the outer region enhanced Col I production.	Mandal et al. (2011a)
	3D printing	Subcutaneous implantation	-	-	Fibrochondrocytes	The scaffold supported to maintain cell phenotype.	Bandyopadhyay & Mandal, (2019)
	Processing into porous matrix	Partial meniscus repair	Meniscectomy	-	-	The scaffold provided a degree of articular cartilage protection, improved tibiofemoral contact pressures.	(Gruchenberg et al., 2014; S. Stein et al., 2019a; S. E. C. Stein et al., 2019b)
	Electrospinning	Partial meniscus repair	Meniscectomy	Sr^2+^	-	The SP-Sr group regenerated the meniscus, which provided better protection to the articular cartilage and slowed down the progression of arthritis.	(Y. Li Y et al., 2020)
Hyaluronic acid	3D printing	Partial meniscus repair	Meniscectomy	-	-	Fibrochondrocyte tissue growed inward and integrated firmly with the surroundings.	Ghodbane et al. (2019b)
	Photocrosslinking	*In vitro* culture	Agarose, gelatin, and PCL	-	Fibrochondrocyte	Cells in MeHA were round, and the ratio of deposited Col II to Col I was close to the value of the inner area region of the native meniscus.	Bahcecioglu et al. (2019b)
	Electrospinning	Subcutaneous implantation	-	-	Fibrochondrocyte	The stiffness of the fibers influenced cell behavior, and cellularity and collagen deposition were greater in the stiffer scaffold.	Song et al. (2020)
Chitosan	Gel casting	*In vitro* culture	Different ratios of chitosan and gelatin scaffolds	-	-	All groups of scaffolds had good meniscal cytocompatibility and the scaffolds conforming to the mechanical strength of the different layers of the meniscus were prepared by different ratios of chitosan and gelatin.	Sarem et al. (2013)
	Crosslinking and dialyzing	Total meniscus repair	PVA/CS scaffold with different seed cells	-	ADSC and AC	Extracellular matrix-rich meniscus tissue was regenerated in all experiment groups, but the meniscus in the AC group had the best protection of the femur and tibia.	Moradi et al. (2017)
Extracellular matrix	Freeze-drying	*Ex vivo* repair model	Different amounts of porcine MDM	-	-	Endogenous meniscal cells and MSCs migrated to the scaffolds, 8% MDM scaffold promoted repair of partial meniscal defects.	Ruprecht et al. (2019)
	Freeze-drying	*Ex vivo* repair model	Meniscus suture	PRP	Fibrochondrocyte	The scaffold promoted cell proliferation and infiltration, generated an amorphous extracellular matrix.	Monibi et al. (2016)
	Freeze-drying	Subcutaneous implantation	Sham-operated	-	-	No sign of inflammation showed on the surrounding of tissues.	Chen et al. (2015)
	Freeze-drying	Total meniscus repair	DCB scaffold, ECM/DCB scaffold	-	-	The ECM/DCB scaffold promoted fibrochondrocyte proliferation and secretion of collagen and GAG, and also promoted meniscal regeneration and prevented cartilage degeneration.	Yuan et al. (2016)
	Thermoresponsive gel	*In vitro* culture	-	-	Chondrocyte, fibroblast	Cell infiltration and proliferation	Wu et al. (2015)
	Thermoresponsive gel	Partial meniscus repair	Collagen scaffold	-	BMSC	ECM scaffolds induced fibrochondrogenesis of BMSCs and enhanced overall healing and cartilage protection of the meniscus	Zhong et al. (2020)

**FIGURE 4:**
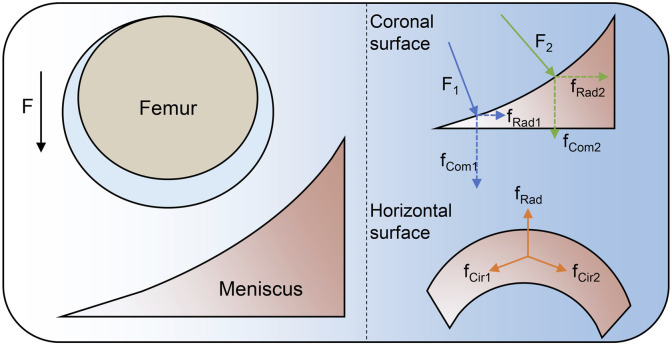
Force analysis of meniscus.

However, homogeneous scaffolds are unable to meet the individualized repair of meniscus, so it is important to design scaffolds that properly guide meniscal repair according to the characteristics of each meniscal department. The fibrous arrangement of the natural meniscus should be simulated so that the meniscal scaffold can resist circumferential and radial stresses in the horizontal direction and compressive stresses in the vertical direction (Figure 4) (Berni et al., 2021). Bahcecioglu et al. 3D printed meniscal scaffolds that mimic the natural collagen arrangement, containing circumferential fibers in the periphery and crossed fibers in the interior. This scaffold exhibited increased compressive and tensile modulus and promoted region-specific protein expression (Bahcecioglu et al., 2019a). In the meantime, well-aligned fibers facilitate the directional migration of cells (Prendergast et al., 2021). In addition, if it involves the repair of the outer two-thirds of meniscus, the scaffold should have a gradient of pores. The gradient reduction of the scaffold pore size from lateral to medial facilitates vascular infiltration, thus allowing the vascular distribution of the repaired tissue to be consistent with that of the natural meniscus (Bahcecioglu et al., 2019a; Guo et al., 2021). We summarize the cases of various natural scaffolds in meniscal injury repair, describe the advantages and disadvantages of structure design of natural material scaffolds, predict the future development direction, and provide ideas for clinical translation of meniscal tissue engineering.

### 3.1 Collagen

Collagen is the most abundant ECM protein, with biodegradability, low immunogenicity, and adequate sources. Collagen self-assembles into cross-striated fibers that provide support for cell growth and are responsible for the mechanical elasticity of connective tissue (Sorushanova et al., 2019). Col I is the major collagen not only in the red-red region of the meniscus (Kwon et al., 2019), but also in mature tendons and ligaments, giving them weight-bearing mechanical properties (Varma et al., 2016). Based on the wide distribution of Col I in living organisms, its easy access to materials, and superior biological properties, it has great potential for meniscal scaffold development.

Meniscal cells derived from different regions, human bone marrow-derived mesenchymal stem cells (BMSCs), synovial cells, and cells from the infrapatellar fat pad were seeded onto aligned electrospun Col I scaffolds and optionally encapsulated in a hydrogel (Baek et al., 2018). Treatment of the scaffolds with transforming growth factor-β1 (TGF-β1) and TGF-β3 increased Col I deposition in all cell types and increased expression of *COL1A1*, cartilage oligomeric matrix protein (*COMP*), Tenascin C (*TNC*), and Scleraxis (*SCX*) genes (Baek et al., 2018). Meniscal cells seeded on collagen scaffolds produced higher levels of Col I than cells seeded on polylactic acid (PLA) (Baek et al., 2016), suggesting a potential advantage of biopolymer scaffolds in promoting collagen deposition. However, the initial mechanical properties of the collagen scaffold may not be as good as the natural meniscus based on the processing technic and the collagenic own characteristic (Meyer, 2019)

The ability of the injured meniscus to repair itself is limited to vascularized areas and is more susceptible to permanent post-traumatic and retrogression in internal non-vascular areas. Human meniscus cells were seeded and cultured on a Col I scaffold for 4 weeks. A collagen scaffold was implanted into a longitudinal tear injury model established in the avascular region of the bovine meniscus and incubated *in vitro* for 3 weeks. Cell-seeded collagen scaffolds showed better integration of natural tissue compared to cell-free collagen scaffolds or the control group (Baek et al., 2016). Histological analysis of *in vivo* animal experiments showed active integration of meniscus-like cartilage into a tissue-engineered biological scaffold in animal models. Active cellular resorption of collagen scaffold decreased over time. Besides, four cases showed mild nonspecific chronic inflammation, and another showed inflammatory engulfment of the scaffold with giant cells at 3 weeks. More importantly, no clinical or histological infections were found in all animal models at any time (Hansen et al., 2013).

Evidence suggests that treatment of meniscal injuries by implantation of a resorbable collagen scaffold may provide more satisfactory clinical outcomes than partial meniscectomy alone (Warth & Rodkey, 2015). The clinical prognosis after CMI implantation is good and relatively stable, particularly relating to restoring knee functions and relieving pain, and the incidence of postoperative complications and reoperations is low (Grassi et al., 2014). When used in combination with concomitant surgeries, such as anterior cruciate ligament reconstruction and high tibial osteotomy, short-term postoperative clinical outcomes improved in invalids who received CMI, with no significant difference between Actifit polyurethane meniscal scaffold and CMI (Houck et al., 2018). Follow-up results at least 10 years after surgery have shown that the implant size usually decreases, but the long-term outcome of the procedure is safe with a low rate of implant failure. Further progression of degenerative knee disease was not observed in most patients (Monllau et al., 2011). Postoperative magnetic resonance imaging (MRI) follow-up results showed an ongoing remodeling with reduced signal intensity and diminished size in CMI. However, since meniscus extrusion remained at the same level, bone marrow edema decreased from 1 year to longer follow-up and appeared to end remodeling at about 5 years after CMI (Schenk et al., 2020). Depending on the histology, new tissue growth to CMI may also be due to the process of synovial overgrowth, but other regenerative mechanisms may also be possible.

**FIGURE 5:**
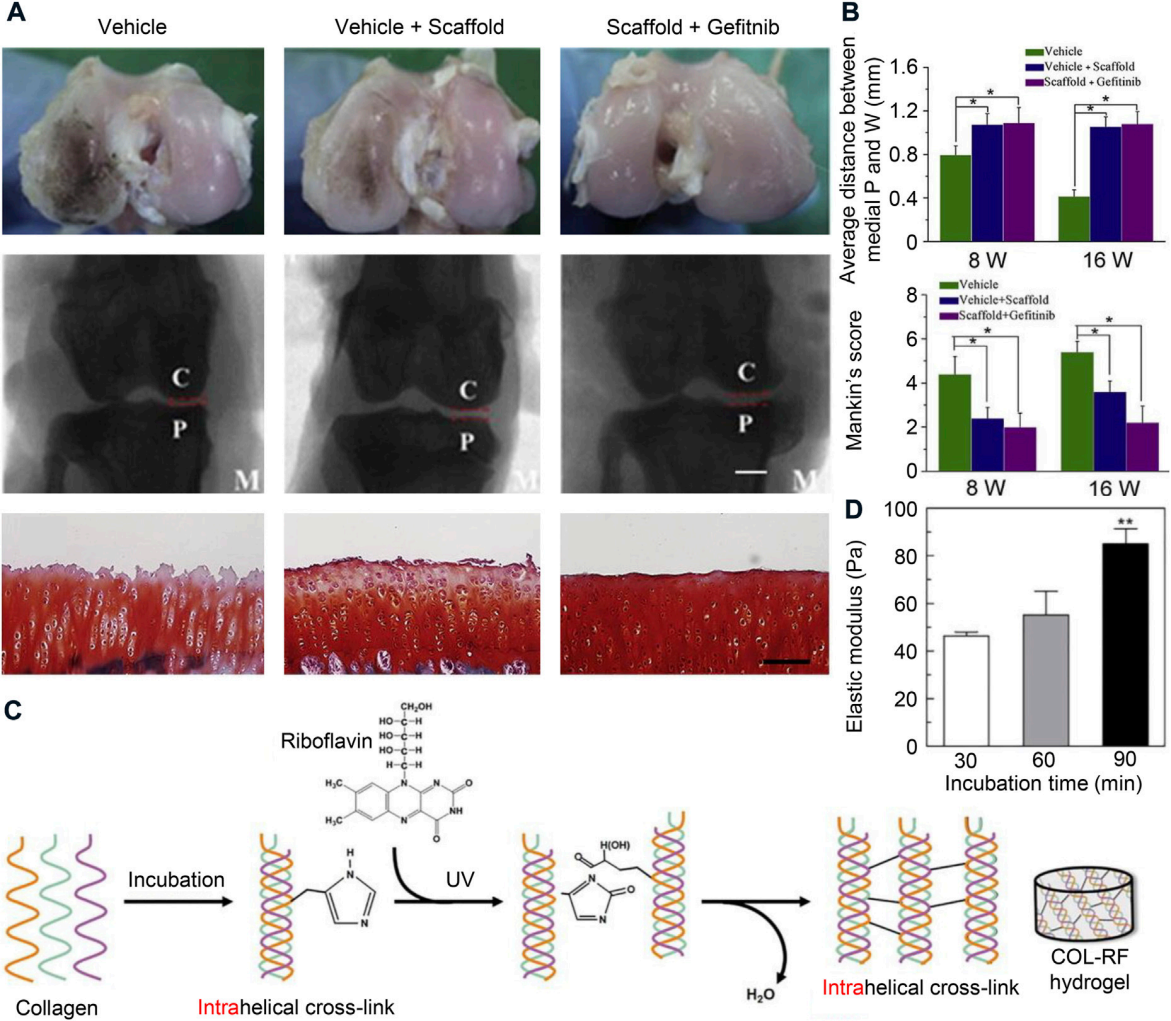
Collagen materials used for meniscus tissue engineering. **(A)** Gross morphology, radiological analysis, and histological staining of the femoral cartilage surface after implantation of collagen scaffold. Bar = 200 μm. C, condyles of femur; P, tibial plateau; M, medial of the knee joint. **(B)** Average distance between medial knee joint, and histological evaluation according to Mankin’s scoring system. Reprinted with permission (Pan et al., 2017). Copyright (2017), Elsevier. **(C)** Synthetic routes of riboflavin-induced photocrosslinked collagen (COL-RF) hydrogel. **(D)** The relationship between elastic modulus and incubation time. Reprinted with permission (Koh et al., 2017). Copyright (2017), Elsevier.

Collagen scaffolds provide strong mechanical strength and effectively promote meniscus regeneration (Pan et al., 2017). However, isolated collagen meniscal scaffolds still have certain limitations of their inherent limitation. For example, the operation and suturing of collagen implants are challenging (Kwon et al., 2019). Because the scaffold has a highly porous surface, which is essential for the differentiation and proliferation of fibrocartilage cells, sutures tend to cut through the implant, making good fixation difficult to achieve. Therefore, most postoperative complications of CMI are due to the suture material rather than the meniscal scaffold (Gwinner et al., 2017). This led us to think about the need to make collagen meniscus scaffolds work better by using a more refined tissue engineering strategy. Since the activation level of epidermal growth factor receptor (EGFR) is in an abnormally high state after meniscal injury, the combination of cell-free collagen scaffold and gefitinib enhances meniscal regeneration (Figures 5A,B A and B) (Pan et al., 2017). Photo-crosslinking using riboflavin and ultraviolet exposure increases the mechanical properties of collagen scaffolds and delays enzyme-triggered degradation of scaffolds (Figures 5C,D) (Heo et al., 2016; Koh et al., 2017). In general, the greatest advantage of collagen is its good biocompatibility, providing a good growth environment for the seed cells, but its lack of processability is also a significant disadvantage, so it needs to be modified or mixed with other materials.

### 3.2 Silk

Silks are natural fibrin polymers (Huang et al., 2018), which embody superior material characteristics and biological functions achieved through a fine hierarchical structure. Through gentle and efficient processing, and careful and rational material design, they can be used to make high-performance, multifunctional, and well-biocompatible materials (López Barreiro et al., 2019). Although a complete study and analysis of the wide variety of silks remain difficult, regardless of the origin, silks are mainly composed of proteins and a few amounts of polysaccharides and lipids (Bhattacharjee et al., 2017). SF is a linear, water-insoluble protein, and could be combined with other biomaterials to form composites (Huang et al., 2018), while sericin is a globular, water-soluble glycoprotein (Chen et al., 2019). The advantages of silk include good biodegradability, biocompatibility, low immunogenicity (Wang et al., 2019), and the competence to remodel *in vivo* and serve on a pattern for natural tissue growth, resulting in vascularization and inward tissue growth.

Silk scaffolds have been widely used for meniscal tissue engineering studies. To generate meniscus-like tissues by *in vitro* experiments, a three-dimensional (3D) silk meniscal scaffold system was designed to mimic the natural meniscal structure by inoculating the scaffold with chondrocytes, human fibroblasts, or adipose-derived stem cells in a spatial separation pattern similar to that of natural tissues (Mandal et al., 2011a; Yang et al., 2017; Ying et al., 2018). The degree of silk scaffold remodeling, tissue inward growth, or other specific cellular behavior can be regulated by increasing growth factors or other signaling factors (Thurber et al., 2015). In a chondrogenic culture in the presence of TGF-β3, cell-seeded constructs increased in cellularity and ECM content. Histology and immunohistochemistry confirmed that having lofty levels of sulfated GAG and type I and II collagen maintained the chondrocyte phenotype (Mandal et al., 2011a). Elevated levels of *COL1A1*, *aggrecan*, and *SOX9* gene expression further confirmed the differentiated and mature cell phenotype (Mandal et al., 2011b). The compressive elastic modulus of the silk scaffold increased significantly with incubation time (Yang et al., 2017). These *in vitro* experiments suggest that porous silk structures can serve as an effective micro-patterned template for endogenous cell migration, proliferation, and differentiation during the fresh meniscus-like tissue forming process.

*In vivo* studies have confirmed that transplantation of different forms of silk products into muscles, skins, bones, or brains showed good biocompatibility (Uebersax et al., 2013; Fernández-García et al., 2016; Ju et al., 2016), causing only low inflammatory responses, such as low infiltration of inflammatory cells, and that these responses were usually transient, this normal response decreases within several weeks after implantation. Depending on the properties of the material and the implantation site, which involves macrophage recruitment and activation, a mild foreign body reaction can result in the formation of multinucleated giant cells (Thurber et al., 2015). All mice were in good condition with no adverse reaction after 4 weeks of subcutaneous transplantation of three types of SF scaffolds, either decellularized, implanted with human meniscus cells, or implanted with human adipose-derived stem cells, into 5-week-old male nude mice (Cengiz et al., 2019). *Bandyopadhyay A et al.* implanted cell-free scaffolds into the subcutaneous abdominal pocket of SD rats. Enhanced degradation and absorption of structures were observed 14 days after implantation of scaffolds compared to day 7, and they had excellent immunocompatibility (Bandyopadhyay & Mandal, 2019). H&E stained images and Masson’s trichrome stained images show that all scaffolds have a high degree of excellent tissue infiltration, angiogenesis, and collagenous ECM formation. And cross-sections show a homogeneous appearance throughout the sample (Cengiz et al., 2019). A partial meniscectomy was operated on the medial meniscus of sheep and an SF scaffold was implanted into the defect. The joint caused no inflammatory reaction 6 months after surgery, and there was no significant difference in cartilage degeneration between the experimental and sham-operated groups. The compression properties of the scaffold were close to those of natural meniscal tissue (Gruchenberg et al., 2014). Nevertheless, the compressive stiffness of the scaffold is greatly increased after implantation, which may interfere with the permanent binding of sents to the meniscal tissue and reduce the chondroprotection of the underlying cartilage (S. E. C. Stein S. et al., 2019; Warnecke et al., 2018). The current study demonstrates the SF scaffold has the attribute of significantly improving tibiofemoral contact pressures, with up to 81% of tibiofemoral contact pressures being transmitted through the meniscus, within the knee after partial meniscectomy (S. Stein S. E. C. et al., 2019). However, the failure of the SF scaffold to fully recapitulate the contact area and pressure of the intact meniscus, especially at high flexion angles, suggests that the biomechanical properties of the scaffold may need further improvements to fully restore tibiofemoral contact mechanics (S. Stein et al., 2019a). It has been demonstrated in friction tests that SF hydrogels have a friction response similar to that of cartilage, dominated by this interstitial fluid support (Parkes et al., 2015). To elucidate the tribological properties of the fibroin meniscal scaffold, the friction of the implant against cartilage and glass was tested. The coefficient of friction between the silk scaffold and cartilage is 0.056, which is greater than the natural meniscus, but less than the threshold required for meniscus replacement (Warnecke et al., 2017).

**FIGURE 6:**
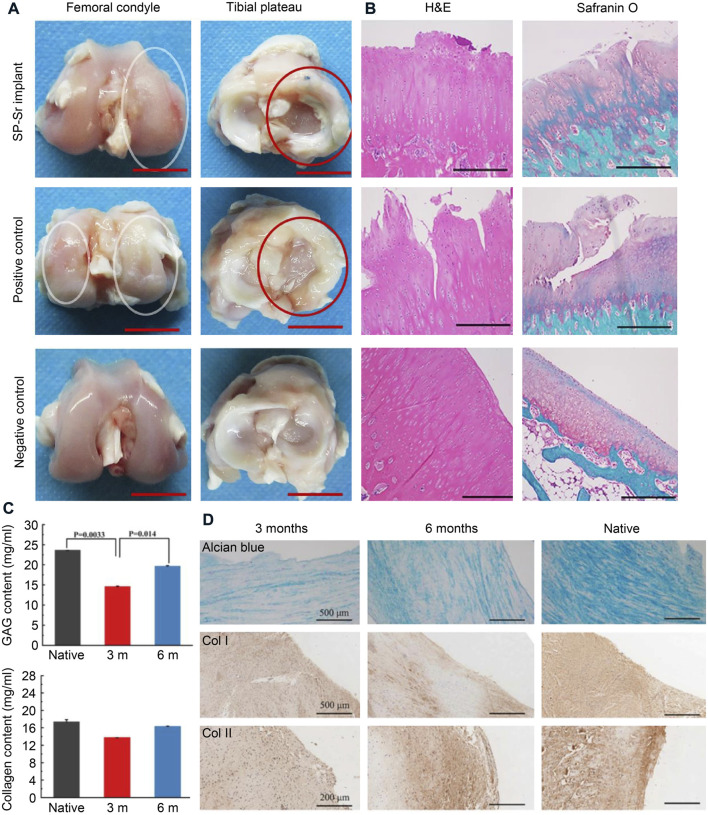
Silk materials used for meniscus tissue engineering. **(A)** Macroscopic observations of regenerated menisci and the corresponding femoral condyles. The red ellipses referred to the meniscus and the white ellipses referred to worn cartilage. Bars = 1 cm. **(B)** H&E and Safranin O-Fast Green staining of articular cartilage surfaces in the femur cartilage. Bars = 500 μm. **(C)** The total GAG and collagen content were estimated in native meniscus and neomeniscus. **(D)** The alcian blue and immunohistochemical staining in native meniscus and neomeniscus. Reprinted with permission (Y. Li Y et al., 2020). Copyright (2017), Elsevier.

Silk fibroin scaffolds still have some shortcomings. The mechanical properties of pure SF scaffolds after freeze-drying are usually unsatisfactory. Fortunately, the biodegradability and mechanical characteristics of SF can be modulated by other suitable polymers (Bandyopadhyay & Mandal, 2019; Y. Li Y et al., 2020; Z. Li Z et al., 2020). For instance, the internally coated collagen enhanced the biocompatibility of the silk sponge, but the outer layer of the collagen would increase the friction properties (Yan et al., 2019). SF and strontium were incorporated with Ɛ-Polycaprolactone by wet-electrospinning method to manufacture the meniscus scaffold, which enhances the secretion of collagen and aggrecan (Figure 6) (Y. Li Y et al., 2020). The combination of silk and cellulose significantly improved the toughness of the hydrogel and promoted the biological activity of fibroblasts (Dorishetty et al., 2020). Compared to collagen, silk proteins are better processed, but silk proteins form β-sheets autonomously, which affect the mechanical properties of the scaffold.

### 3.3 Hyaluronic acid

Hyaluronic acid (HA) is a GAG consisting of the repeating disaccharide portion of d-glucuronic acid and N-acetyl-d-glucosamine and is found in large amounts in the skin and musculoskeletal tissues. Hyaluronic acid has been widely used in tissue regeneration and medicine due to its superior physiological function and biocompatibility. It also has a well-controlled degradation rate and mechanical properties. HA alone has a relatively fast degradation rate and weak mechanical strength, and the most common modification method is the reaction of methacrylic anhydride with HA to form methacrylated hyaluronic acid (MeHA), which has photocrosslinking properties (Kenne et al., 2013). The photocrosslinked HA has a slower degradation rate and higher mechanical stage and is related to the degree of methyl substitution (D'Amora et al., 2018). *Zhang et al.* obtained a series of photocurable hydrogels with different degrees of crosslinking by adding different concentrations of the water-based tetrathiol crosslinker (PE(NAC)4) to MeHA, which had different gelling time, mechanical strengths, and degradation rates (Zhang et al., 2020). In addition, HA has functional groups that can be chemically modified, carboxyl and hydroxyl. The carboxyl of glucuronic acid is a good target for 1-ethyl-3- [3-(dimethylamino)-propyl]-carbodiimide (EDC) mediated amide and ester formation of HA (Knopf-Marques et al., 2016). *Kim et al.* covalently bound tyramine (TA) to hyaluronan by EDC and mixed it with gelatin to obtain hydrogels mediated by tyrosinase (TYR) cross-linking. Compared to gelatine alone, mixtures of HA and gelatine in the presence of TYR have faster gel transformation time and higher mechanical properties, and the mechanical peculiarities and degradation kinetics were regulated by TA substitution and TYR concentration (Kim et al., 2018).

HA and its modifications have been widely used to repair with good results. Intra-articular injection of high molecular weight HA has a positive therapeutic effect on osteoarthritis or acute cartilage injury, and it regulates the expression of a variety of mi-RNAs that are associated with cartilage degeneration, apoptosis, and inflammation (Genemaras et al., 2018). However, viscosupplementation therapy prevents macroscopic damage to the meniscus but does not recover its collagen fibrous tissue and viscoelasticity (Levillain et al., 2017). However, cell proliferation and migration were observed when HA was added to the culture medium to stimulate meniscal cells. Not only that, but when the injured meniscal tissue was cultured in cultures containing HA, the accumulation of collagen II around the tear was also observed, but collagen I accumulation around the meniscal injury site was not induced (Tanaka et al., 2017). Thus, the use of HA as raw material and the application of tissue engineering methods to repair damaged meniscus have become a recent research hotspot. *Ghodbane et al.* injected collagen HA sponge as an extracellular matrix into the meniscal scaffold, which matched the circumferential tensile and axial compressive properties of the natural meniscus and had appropriate porosity (Ghodbane et al., 2019a). At 24 weeks after implantation, inward cell growth was observed, producing dense tissue liked fibrocartilage with significant collagen and GAG deposition. On the downside, some of the scaffolds were displaced postoperatively, resulting in low meniscal repair and loss of meniscal protective effect on articular cartilage (Ghodbane et al., 2019b). *Bahcecioglu et al.* inoculated porcine fibrocartilage cells with agarose, methacrylated gelatin (GelMA), and MeHA, hybrid hydrogels, and 3D printed PCL scaffolds, and then evaluated the potential for *ex vivo* meniscal regeneration under dynamic compression. The results showed that hydrogels had a higher potential for meniscal regeneration compared to PCL, with agarose and MeHA favoring the regeneration of the inner meniscal region and gelatin favoring the regeneration of the outer meniscal region. (Bahcecioglu et al., 2019b). *Song et al.* synthesized soft and stiff MeHA scaffolds by electrostatic spinning, in which stiffness was varied by the degree of MeHA cross bonding. The adhesion and migration of meniscal fibrochondrocytes (MFC) to the fibermesh were also investigated, in which the softer MeHA fibermesh was susceptible to deformation and densification by cell traction, whereas the stiffer MeHA fibrous network supported more MFC invasion when placed near the meniscal tissue (Song et al., 2020). In conclusion, the use of HA scaffolds for tissue repair is a promising approach, and enough investigation has been done on this material, yet its use in meniscal repair is often used as an adjunct to synthesis (Halili et al., 2014; Heo et al., 2016). Most notably because of its good biocompatibility but weak mechanical strength, although the suitability of HA has been enhanced by various modifications, the potential damage of these modifiers to the tissue has yet to be investigated.

### 3.4 Chitosan

Chitosan (CS) is a biopolymer made by deacetylation of chitin with a structure similar to GAG. CS has a wide range of applications in biomedical fields due to its splendid biodegradability, bioactivity, and biocompatibility. CS can provide active sites for cell adhesion, cell-cell, and cell-matrix interactions, which in turn plays an important role in regulating cell proliferation and function. The cationic property of CS allows for the immobilization of negatively charged enzymes, proteins, GAGs, polysaccharides, or other negatively charged molecules under mildly acidic conditions (Sultankulov et al., 2019). CS can also form interconnected porous structures after lyophilization and allows for large numbers of cells to be inoculated at once, improving cell migration and proliferation. The *in vivo* degradation of CS also shows synergistic effects on the long-term performance of tissue engineering cells by affecting many cellular processes such as cell growth, tissue regeneration, and host response (Ahmed et al., 2018). The molecular chain of CS contains reactive amino and hydroxyl making it susceptible to chemical modifications. Combining the major ECM molecules, collagen and chondroitin-6-sulfate, onto a hyaluronic acid/chitosan multilayer can be used as a bionic surface for meniscus repair to reverse meniscus cell dedifferentiation (Tan et al., 2010).

Nevertheless, the properties of isolated CS scaffold materials have some limitations in the preparation of meniscal scaffolds, and the modification of CS is currently a hot spot for research in the field of biomaterials. *Sarem M et al.* prepared chitosan/gelatin composite scaffolds exhibiting highly inter-connected pore architecture with swelling and degradation behavior using genipin as a biocompatible cross-linking agent. Gelatin improves the biocompatibility of CS, and gelatin/chitosan scaffolds have excellent compatibility with meniscus-derived cells (Sarem et al., 2013). In addition, the chitosan/calcium polyphosphate composite scaffold enhances the necessary mechanical properties of the meniscal scaffold. The degradation rate of the scaffold can be controlled by varying the amount of calcium polyphosphate to avoid premature degradation and ensure that the scaffold is filled with new tissue. *Moradi et al.* produced a polyvinyl alcohol/chitosan scaffold that compared the meniscal repair effects of adipose-derived stem cells and articular chondrocytes. The scaffold inoculated with chondrocytes promoted meniscal regeneration well and improved the mechanical properties of the regenerated meniscal tissue (Moradi et al., 2017).

Overall, CS has good properties and is a significant element of scaffold material research and one of the future research directions in the field of biomaterials. Researchers are continuously exploring the potential of CS as a biomaterial, aiming to eliminate the limitations of existing materials. New CS derivatives and composites could certainly make it an ideal scaffold material.

### 3.5 Extracellular matrix

ECM is acellular component of tissues that provides scaffolding for cells and regulates normal tissue morphogenesis, differentiation, matrix renewal, and dynamic homeostasis (Swinehart & Badylak, 2016). Bioactive molecules that maintain tissue homeostasis and promote regeneration are present in ECM and can act through a range of cell-cell and cell-matrix interactions (Watt & Huck, 2013; Monibi & Cook, 2017). The tissue-derived stroma has a guiding function in the differentiation of stem cells (Rothrauff et al., 2017; Yuan et al., 2017). A specific comparison of the effects of meniscus-derived ECMs (mECMs) on hBMSCs in different regions showed that internal mECMs promoted fibrocartilage differentiation of hBMSCs, whereas external mECMs enhanced more fibroblast phenotypes (Shimomura et al., 2017). The meniscus of rats, sheep, pigs, and humans can be decellularized using numerous techniques, including physical, (freeze-thawing and sonication), chemicals (detergents, EDTA, and hypotonic buffers), biological (enzymatic digestion), and their combinations (Chen et al., 2017; Monibi & Cook, 2017). *In vitro* experiments are remarkable for testing the performance of ECM meniscal scaffolds. Porcine meniscus-derived matrixes (MDM) were prepared and tested for their effectiveness in promoting meniscal repair by migrating endogenous meniscus cells from the surrounding meniscus or exogenously seeded hBMSCs. Both endogenous meniscus cells and hBMSCs permeated into the MDM scaffold. In the absence of exogenous cells, 8% of the MDM scaffold promoted the overall repair of meniscal defects *in vitro* (Ruprecht et al., 2019). *In vitro* experiments to assess cytotoxicity and cytocompatibility of ECM meniscal scaffold, synovial cells, and meniscal chondrocytes were seeded onto decellularized meniscal scaffolds, after 8 days of incubation under basic conditions, the scaffold was not cytotoxic to either cell. In an *in vitro* model of meniscal repair, ECM-derived scaffolds were inoculated into excised menisci and cultivated for 42 days, and histological evaluation showed normal tissue architecture and cellularity of meniscus (Monibi et al., 2016). In addition, the scaffolds were implanted subcutaneously into rats for 7, 14, and 28 days. No inflammatory cells were detected to infiltrate the implant. The structure of the scaffold was stable for more than 14 days. Absorption and turnover of the scaffolds were observed on day 28. The tissue around the implant showed no signs of inflammation and was comparable to the sham operation control group (Chen et al., 2015).

**FIGURE 7:**
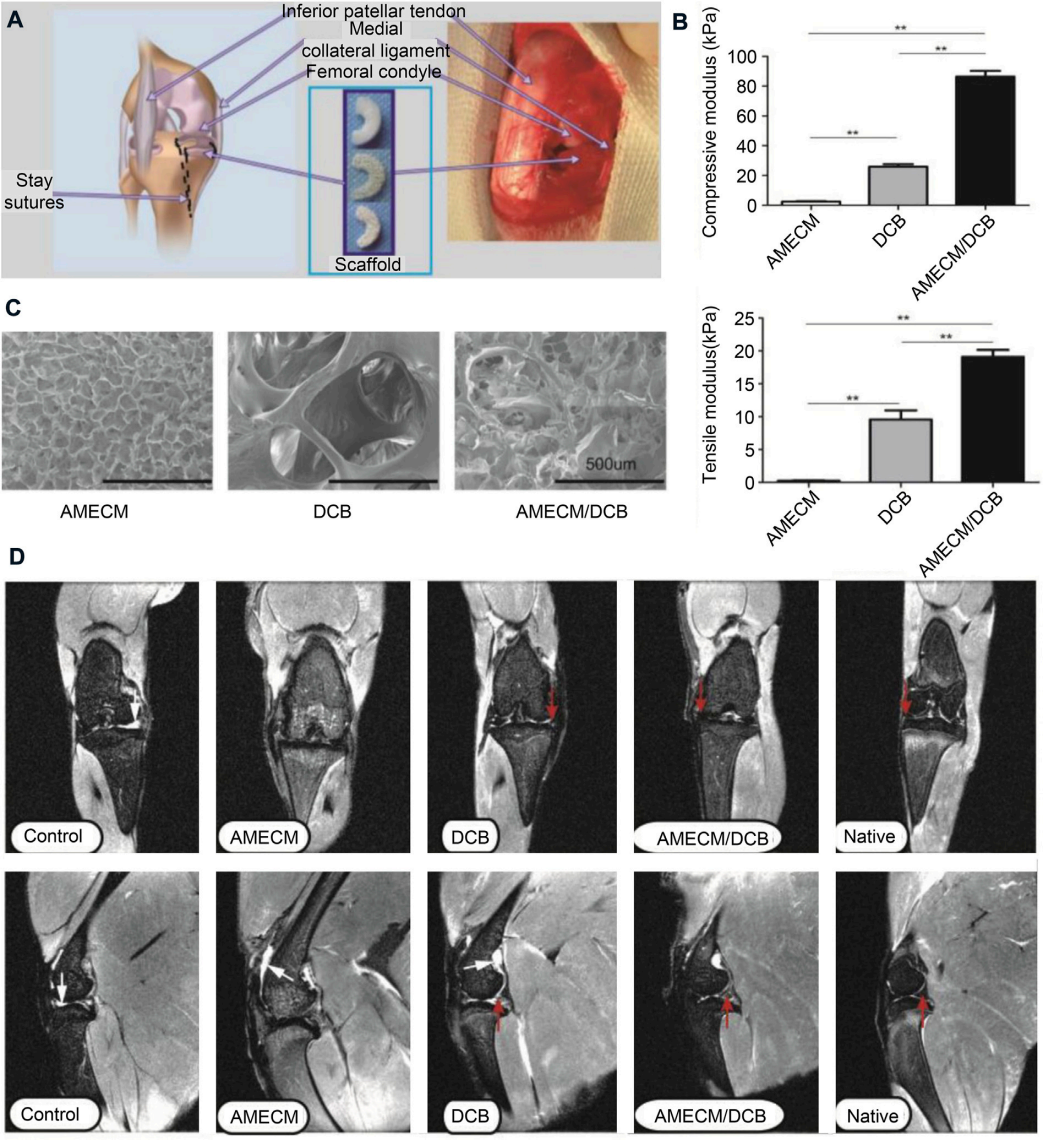
Extracellular matrix used for meniscus tissue engineering. **(A)** Surgical strategies for repairing meniscus with extracellular matrix scaffolds. **(B)** Comparative mechanical modulus of the different scaffolds. **(C)** Scanning electron micrographs of the different scaffolds. **(D)** Magnetic resonance imaging (MRI) of rabbit knees 6 months after surgery. The red arrow indicates the regenerated menisci. Reprinted with permission (Yuan et al., 2016). Copyright (2016), Elsevier.

The intermediate region of the bovine meniscus is an excellent material for the preparation of ECM scaffolds. The collagenous fibrils of the meniscal fibrocartilage in the middle zone become more highly oriented perpendicular to the direction of compression (Sizeland et al., 2020). The decellularized porcine medial meniscus maintains the tensile biomechanical properties of the native meniscus but has lower elastic moduli in tension and compression compared to the native meniscus. These changes in biomechanical properties may be due to the reduction of GAG content during decellularization (Abdelgaied et al., 2015). GAG plays a significant role in water regulation within the meniscus. Although some compositional changes in the ECM are to be expected during processing, it is clear that numerous essential structural components remained functional while maintaining biomechanical properties (Stabile et al., 2010). Demineralized cancellous bone (DCB) is an extensively used bioactive scaffold for tissue engineering with a natural 3D porous structure and pretty biocompatibility (Wang et al., 2016). Acellular meniscus extracellular matrix (AMECM) and DCB were used to construct different types of triaxial porous meniscal scaffolds, and the AMECM and DCB played a good synergistic repair effect on the meniscus (Figure 7) (Yuan et al., 2016).

Injectable hydrogels can be derived from tissue-specific ECM, thus providing a bionic environment for cell delivery and allowing seamless regeneration of tissue defects (Yuan et al., 2017). An injectable ECM hydrogel material was developed in the porcine meniscus. This meniscus-derived ECM hydrogel displays a fibrous morphology with adjustable compressive force and initial modulus. The hydrogel promoted the growth of bovine chondrocytes and mouse 3T3 fibroblasts, showing good biocompatibility. Furthermore, the subcutaneous implantation dedicated that the hydrogels were favorable for cell infiltration. (Wu et al., 2015). Decellularized ECM retains essential proteoglycans and collagen and encapsulates BMSCs in ECM or hydrogels. When applied to an orthotopic model of meniscal injury in SD rats, ECM outperformed collagen I scaffold in reducing bone redundancy formation and preventing narrowing of joint space and osteoarthritis development, as demonstrated in histological and micro-CT analyses(Zhong et al., 2020). Although the mECM had higher concentrations of TGF-β and bFGF (Rothrauff et al., 2017), mECM hydrogels did not significantly support fibrochondrogenesis of hMSCs in the absence of TGF-β3, with a decrease in cell numbers and OHP content of hydrogels, and the lack of sulfated GAG production (Yuan et al., 2017).

However, the traditional AMECM preparation process ignores the regionality of the meniscus and processes the entire meniscus into a homogeneous scaffold. It is important to maintain the regional specificity of meniscus. Yun et al. decellularized the inner cartilage region, transition region, and outer fibrous region of the porcine meniscus, respectively, to prepare a meniscal scaffold with a composition gradient. After this scaffold was implanted *in vivo*, the expression of region-specific proteins was induced, with a large amount of GAG secreted on the inner side and fibrin mainly on the outer side (Yun et al., 2021). Therefore, ACECM is also an ideal material for meniscal tissue engineering, not only for its biocompatibility and ability to promote stem cell differentiation, but also for its ability to be processed into structurally controlled scaffolds through various techniques.

## 4 Future directions and conclusion

There is no perfect method for meniscus injury in clinical practice. Meniscectomy removes the damaged part of the meniscus, which to some extent solves the patient’s pain and joint interlocking symptoms, but it may cause direct contact with articular cartilage, leading to more serious cartilage wear, and eventually osteoarthritis. Meniscus suture surgery requires very high postoperative rehabilitation for patients. Patients who have undergone this procedure need to strictly control the angle of joint flexion and extension, and the timing of lower limb weight-bearing. For patients with large meniscus injuries, if they have stable, correctly aligned joints and are in the early or earlier stages of arthritis, it is best to use meniscus replacement. Although it can significantly improve the prognosis of patients, the source of the allogeneic meniscus is very scarce, and artificial meniscus may become the key to meniscus injury repair. A large number of artificial meniscuses have been developed, among which natural material meniscus prosthesis accounts for a large proportion due to its good biocompatibility and biological activity. Various natural materials have their advantages and disadvantages, decellularized ECM is taken from the natural meniscus, and by partitioning decellularization, a scaffold with a highly similar structure to the natural meniscus can be prepared, but the clinical translation must face ethical review; collagen and hyaluronic acid are also components of the organism and are biocompatible, but they need to be modified or mixed with other polymers to improve processability; well-processed silk proteins and chitosan are also ideal materials for meniscal scaffolds, but they are not mammalian in origin and may cause inflammatory reactions in the organism.

Although many kinds of natural materials have been developed to repair the meniscus and achieved some gratifying results. But it is undeniable that there is still a long way to go before the clinical transformation of natural materials meniscus tissue engineering. Natural materials have advantages over synthetic materials in terms of access, biocompatibility, degradation, etc. As the therapeutic power of seed cells is gradually explored, more accurate and efficient loading of seed cells will play a more important role in tissue engineering, and natural materials are ideal carriers for seed cells. With advances in 3D printing technology, such as the development of stereo lithography appearance printing and bio-ink printing, the processing performance of natural materials has also improved considerably. Although it still does not reach the level of controllability of synthetic materials, the mixture of natural and synthetic materials is also an effective way to combine the advantages of both, such as synthetic materials as a skeleton and natural materials as a filler. Therefore, natural materials are ideal for meniscal tissue engineering, but how to improve natural materials for better repair and higher efficiency is still an important trend for natural materials in the future.

Then, the complex regionalized structure of the meniscus is also one of the difficulties of repair. The white-white area of the meniscus has no blood vessels, so its inherent repairability is very poor. This also leads to a defect in this area that cannot be repaired well even in the presence of the meniscus scaffold. This requires future research to focus on the repair of the white-white area. Perhaps finding clues for the specific differentiation of stem cells into the chondrocyte-like cell of the white-white area is the key to the white-white area repair, including mechanical stimulation, differentiation factors, and special arrangements of materials, etc. The red-white area is the junction of the red-red area and the white-white area. The cell phenotype and collagen arrangement gradually change in this area, so it has the function of two areas in meantime. In the face of meniscus damage that spans the red-white zone, reconstructing the gradient of cells and collagen in this area is conducive to the integration of tissues and surroundings, better transmitting the stress on the meniscus, and transforming radial force into circumferential force, to avoid stress concentration. The red-red area is the area rich in blood vessels. The main point of repair in this area is to expand the porosity of the scaffold as much as possible under the premise of ensuring the mechanical strength, to facilitate the penetration and metabolism of nutrients.

As for the entire replacement of the meniscus, it is still a difficult problem. As mentioned above, the three areas of the meniscus have different repair points. Therefore, it is necessary to meet the repair requirements of the three areas on a scaffold. It is best to include some gradient signals that simulate the natural meniscus. Future research directions should design pore size gradients to influence the infiltration of meniscal vessels and precisely replicate the vessels distribution; mechanical strength gradients should also follow the force analysis of the natural meniscus to simulate the mechanical properties of each region, resulting in a collagen arrangement consistent with the natural structure; or load gradient inducing factors to promote regional differentiation of stem cells to form functionally compatible tissues.

In addition, inflammatory and oxidative reactions from meniscal injuries and implantation cannot be ignored, and attention to the immune microenvironment of injured area is an important future direction. Natural materials have good biocompatibility and degradation products can participate in the normal metabolism process, so they won’t cause excessive inflammation or oxidative reactions. In addition, although inflammatory and oxidative responses do not persist throughout the meniscal repair process, modulating the immune microenvironment and terminating the inflammatory response at the right time can improve the efficiency of meniscal repair. A good transition between anti-inflammation and repair can be achieved by releasing anti-inflammatory and inducing agents in a consistent sequence, and the difference in material degradation rates can also be used to construct an integrated anti-inflammatory-repair meniscal scaffold.

In conclusion, the research on the tissue engineering of the meniscus needs to be further in-depth, mainly including the following aspects: 1) Continuing to optimise the materials used in the preparation of meniscal scaffolds; 2) A bionic integrated meniscus prosthesis based on the differences in the various regions of the meniscus; 3) In-depth study of the mechanism of differentiation of stem cells into different cell phenotypes of the meniscus (mechanical stimulation, differentiation factors); 4) Observe the long-term repair effect of the meniscus scaffold implantation.

## Abbreviations

3D, three dimension; AC, articular chondrocyte; ADCS, adipose- derived stem cell; BMSC, bone mesenchymal stem cells; Col, collagen; CS, chitosan; DCB, demineralized cancellous bone; ECM, extracellular matrix; GAG, glycosaminoglycan; IPFP, infrapatellar fat pad; MDM, meniscus-derived matrix; PCL, polycaprolactone; PRP, platelet riched plasma; PVA, polyvinyl alcohol; TGF-β, transforming growth factor-β; TMSC, tonsil-derived mesenchymal stem cells.
